# Supplementary material for: Spin current generation and relaxation in a quenched spin-orbit-coupled Bose-Einstein condensate
Source: Nat Commun. 2019 Jan 22;10:375. doi: 10.1038/s41467-018-08119-4 (PMC6343014; doi:10.1038/s41467-018-08119-4)
Supplement: Supplementary file 1 — Supplementary Information [file 41467_2018_8119_MOESM1_ESM.pdf]

Supplementary Information: Spin Current Generation and Relaxation in a Quenched Spin-Orbit Coupled Bose-Einstein Condensate

Chuan-Hsun Li,[1] Chunlei Qu,[2,3,4] Robert J. Niffenegger,[5,*] Su-Ju Wang,[5,†] Mingyuan He,[6] David B. Blasing,[5] Abraham Olson,[5] Chris H. Greene,[5,7] Yuli Lyanda-Geller,[5,7] Qi Zhou,[5] Chuanwei Zhang,[2] and Yong P. Chen[5,1,7,‡]

[1]*School of Electrical and Computer Engineering, Purdue University, West Lafayette, Indiana 47907, USA*
[2]*Department of Physics, The University of Texas at Dallas, Richardson, Texas 75080, USA*
[3]*INO-CNR BEC Center and Dipartimento di Fisica, Università di Trento, Povo 38123, Italy*
[4]*JILA and Department of Physics, University of Colorado, Boulder, Colorado 80309, USA*
[5]*Department of Physics and Astronomy, Purdue University, West Lafayette, Indiana 47907, USA*
[6]*Department of Physics, Hong Kong University of Science and Technology, Clear Water Bay, Hong Kong, China*
[7]*Purdue Quantum Center, Purdue University, West Lafayette, Indiana 47907, USA*
(Dated: December 6, 2018)

Supplementary Note 1: Control Experiments

Dipole oscillations of a SO coupled BEC with a single dressed spin component in the $|\downarrow'\rangle$ state. By quickly changing the Raman coupling/detuning as in [1], we can apply a synthetic electric field to a BEC with a single dressed spin component to excite its dipole oscillations in the optical trap. The experimental timing diagram is similar to Fig. 1b in the main text. First, an 80 ms ramp is used to achieve an initial Raman coupling $\Omega_{\rm I} = 3.7\ E_{\rm r}$, where the initial detuning in this case is chosen such that the band is tilted and only $|\downarrow'\rangle$ is present. Subsequently, both $\Omega_{\rm I}$ and the initial detuning are held for another 100 ms. Then, $\Omega_{\rm I}$ is changed to $\Omega_{\rm F}$ while the initial detuning is changed to $\delta_{\rm R} = \delta'(\Omega_{\rm F}, \varepsilon)$ (which realizes a balanced band at $\Omega_{\rm F}$) in 1 ms. This gives a spin current with a net mass current generated from a single dressed spin component. For example, Supplementary Fig. 1 shows the dipole oscillations of a dressed BEC in the $|\downarrow'\rangle$ state at $\Omega_{\rm F} = 1.0\ E_{\rm r}$ and $\delta_{\rm R} = \delta'(\Omega_{\rm F}, \varepsilon)$ (such that the double minima in the ground dressed band are balanced, although only the one corresponding to $|\downarrow'\rangle$ is occupied). Such single-component dipole oscillations are observed to possess very little damping ($1/Q < 0.05$) and without noticeable thermalization within the time scale of the experiment (30 ms), similar to the work in [1]. Similar results are obtained for the measurements performed at different $\Omega_{\rm F}$, as shown in the red square data in Fig. 3f in the main text. Note that in the TOF images, the dominant bare spin component is $|\downarrow\rangle$ (red). There is a minority component in $|\uparrow\rangle$ oscillating in phase with but is $2\hbar k_{\rm r}$ away from $|\downarrow\rangle$, and thus is not shown in Supplementary Fig. 1. The control experiment shows that without a collision partner (i.e. the other dressed spin component in the $|\uparrow'\rangle$ state), the dipole oscillations of a single dressed spin component is very weakly damped without noticeable thermalization within the time scale of the experiment.

Common-mode dipole oscillations of two dressed spin components of a SO coupled BEC. We also excite common-mode dipole oscillations of two dressed spin components of a SO coupled BEC with equal populations in $|\uparrow'\rangle$ and $|\downarrow'\rangle$ (Supplementary Fig. 2) by ramping the optical trap power up and back down in 1 ms. This applies the same force to both dressed spin components and actuates their in-phase dipole oscillations in the trap, creating a mass current without a spin current, therefore also no collisions between the two dressed spin components. To analyze the momentum damping of the individual atomic cloud, $\hbar k_\uparrow$ or $\hbar k_\downarrow$ is fitted to a damped sinusoidal function to obtain the corresponding $1/Q$. We find that such common-mode dipole oscillations in the trap are very weakly damped ($1/Q < 0.05$) without noticeable thermalization within the time scale of the experiment (30 ms). This shows that SOC alone would not cause momentum damping of the individual atomic cloud if there is no relative collision between the two spin components.

---

* Current address: Lincoln Laboratory, Massachusetts Institute of Technology, 244 Wood Street Lexington, MA 02421, USA
† Current address: J. R. Macdonald Laboratory, Department of Physics, Kansas State University, Manhattan, Kansas 66506, USA
‡ yongchen@purdue.edu

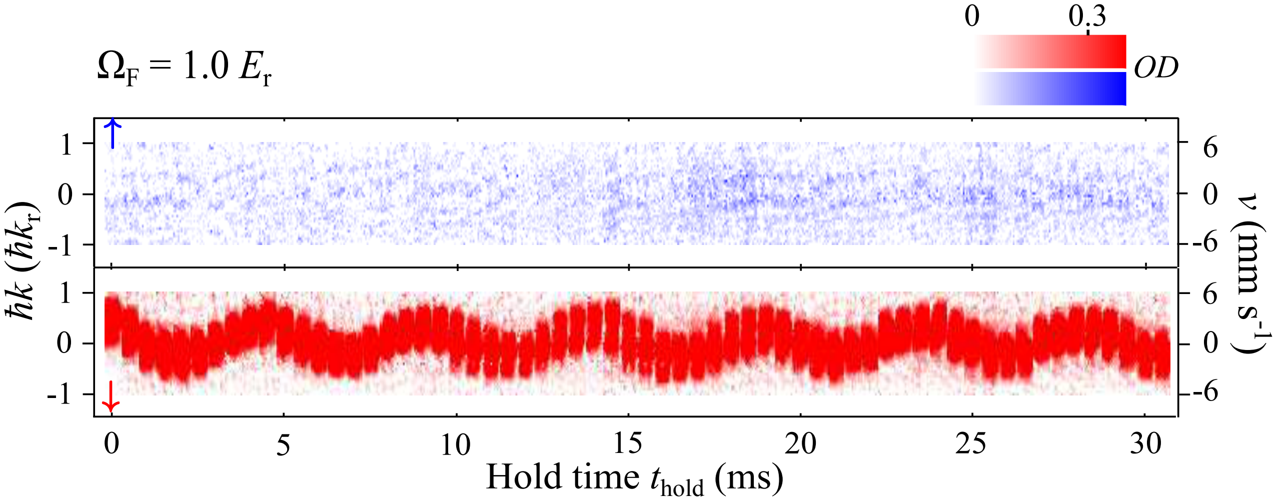

Supplementary Figure 1. **Dipole oscillations of a BEC with a single dressed spin component in $|\downarrow'\rangle$.** Combined TOF images vs t_{hold} for a dressed BEC in $|\downarrow'\rangle$ ($\Omega_F = 1.0\ E_r$, $\delta_R = \delta'(\Omega_F, \varepsilon)$, shown in the main text Fig. 3h) undergoing dipole oscillations, showing very weak damping ($1/Q < 0.05$) and negligible thermalization. Each slice in the image shown is a TOF image at a given t_{hold}, but compressed along the horizontal direction. The vertical axis shows the mechanical momentum $\hbar k$ of atoms. The time step between successive image slices is 0.5 ms. The figure shows bare spin components $|\downarrow\rangle$ in red and $|\uparrow\rangle$ in blue plotted in the lower and upper panels, respectively.

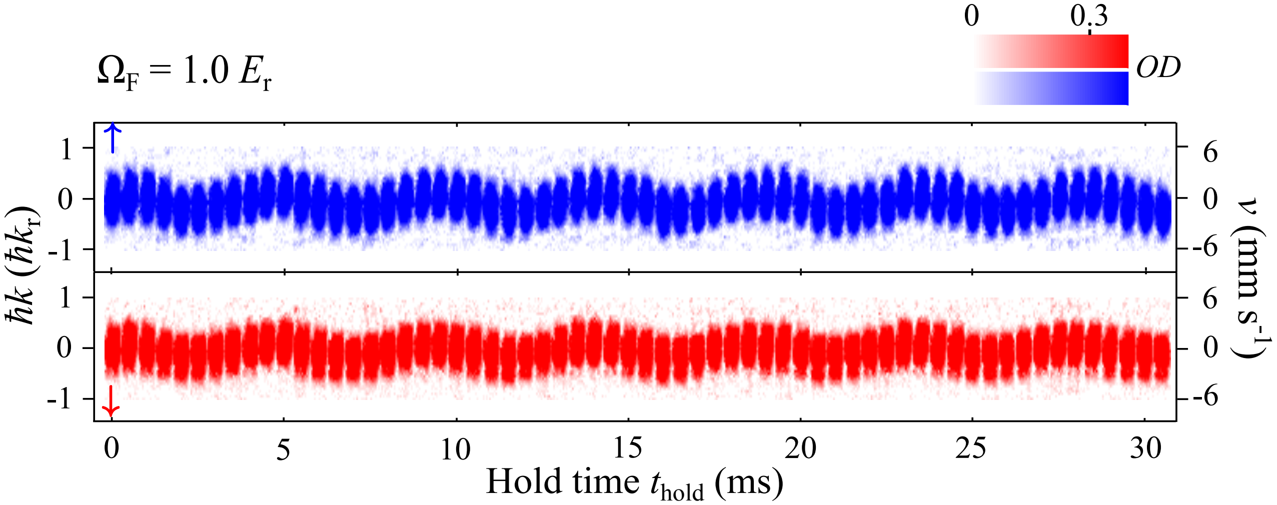

Supplementary Figure 2. **Common-mode dipole oscillations of two dressed spin components of a SO coupled BEC.** Combined TOF images vs t_{hold} for two dressed spin components of a SO coupled BEC with equal populations in $|\uparrow'\rangle$ and $|\downarrow'\rangle$ ($\Omega_F = 1\ E_r$, $\delta_R = \delta'(\Omega_F, \varepsilon)$) undergoing in-phase dipole oscillations, showing very little damping ($1/Q < 0.05$) with negligible thermalization. Each slice in the image shown is a TOF image at a given t_{hold}, but compressed along the horizontal direction. The time step between successive image slices is 0.5 ms. The figure shows $|\downarrow\rangle$ in red and $|\uparrow\rangle$ in blue plotted in the lower and upper panels, respectively.

Supplementary Note 2: Observation of the $m = 0$ Quadrupole Mode of a Dressed BEC with Another Set of Trap Frequencies.

To further verify the excitation of the $m = 0$ quadrupole mode in the dressed case, we intentionally changed the trap frequencies to $\omega_z \sim 2\pi \times (21 \pm 3)$ Hz and $\omega_x \sim \omega_y \sim 2\pi \times (144 \pm 10)$ Hz, and measured the aspect ratio of the condensate as a function of $t_{\rm hold}$ at $\Omega_{\rm F} = 1.3\ E_{\rm r}$ (Supplementary Fig. 3a, with select TOF images shown in Supplementary Fig. 3b) with all the other experimental parameters similar to Fig. 5f in the main text. The data after the dashed line ($t_{\rm hold} \sim 2\tau_{\rm damp}$) is fitted to a damped sinusoidal function. The extracted aspect ratio oscillation frequency is around 34 Hz, again consistent with the prediction $f_m = \sqrt{2.5}\omega_z/(2\pi) \sim 33$ Hz for the $m = 0$ quadrupole mode. This confirms the excitation of the $m = 0$ quadrupole mode after the SDM is damped out in the dressed case.

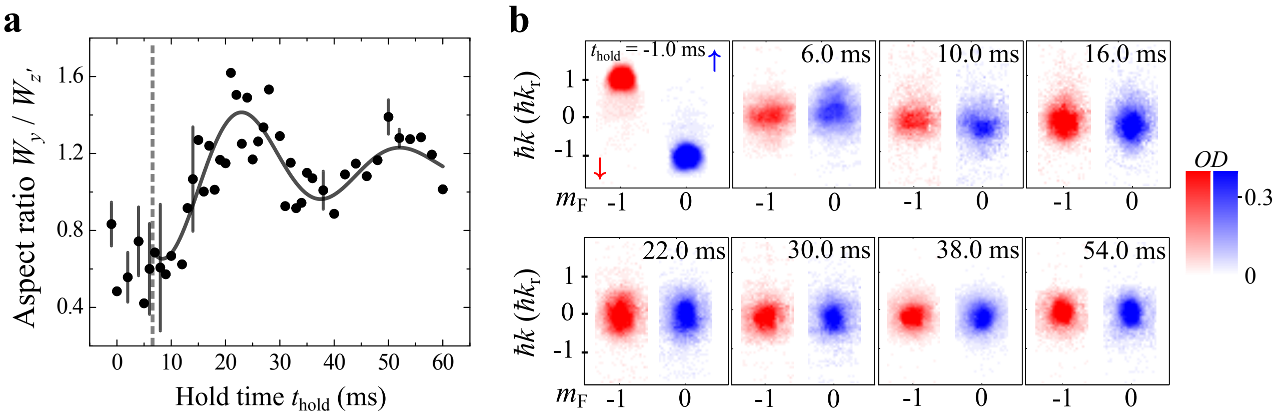

Supplementary Figure 3. **Observation of the quadrupole mode of a dressed BEC with another set of trap frequencies.** (a) For $\Omega_{\rm F} = 1.3\ E_{\rm r}$ with trap frequencies $\omega_z \sim 2\pi \times (21 \pm 3)$ Hz and $\omega_x \sim \omega_y \sim 2\pi \times (144 \pm 10)$ Hz used in this measurement, the observed aspect ratio oscillation frequency is around 34 Hz, consistent with the expected $m = 0$ quadrupole mode frequency $f_{m=0} = \sqrt{2.5}\omega_z/(2\pi) \sim 33$ Hz. This further verifies the excitation of the $m = 0$ quadrupole mode. The oscillation frequency is obtained using a damped sinusoidal function to fit the data following the SDM is damped out (when $t_{\rm hold} \sim 2\tau_{\rm damp}$ as indicated by the dashed line). The representative error bars are standard deviation of at least three measurements. (b) Select TOF images are typically the average of a few repetitive measurements.

Supplementary Note 3: Control Simulations, Phase of BEC Wavefunctions in SDM, and Movies

Effect of immiscibility on SDM. We have used GPE simulations for the bare case with intentionally modified interactions to study the effect of immiscibliity on the SDM. Supplementary Fig. 4 shows the damping of the relative momentum $\hbar k_{\rm spin}$ of the SDM for 5 cases without and with modified interactions. Case 1 is the original bare case without modification of interactions, with the intraspecies and interspecies interaction parameters g_{ii} and g_{ij} ($i, j =\uparrow, \downarrow$ and $i \neq j$) given by Eqs. (18,19) in the main text, respectively. Case 2 corresponds to the same intraspecies interaction parameter $\tilde{g_{ii}} = g_{ii}$ and a modified interspecies interaction parameter $\tilde{g_{ij}} = 1.5g_{ij}$. Case 3 corresponds to $\tilde{g_{ii}} = 1.5g_{ii}$ and $\tilde{g_{ij}} = 1.5g_{ij}$. Case 4 corresponds to $\tilde{g_{ii}} = 1.5g_{ii}$ and $\tilde{g_{ij}} = g_{ij}$. Case 5 corresponds to $\tilde{g_{ii}} = 1.8g_{ii}$ and $\tilde{g_{ij}} = g_{ij}$. Such modification of interactions is done by immediately increasing the interaction g-parameters to the desired values as soon as Ω is changed from $\Omega_{\rm I}$ to $\Omega_{\rm F}$. Among all the cases, only case 2 is immiscible and we observe that case 2 possesses the strongest damping, thus suggesting that immiscibility is particularly effective to enhance the damping of the SDM. This is further supported by the observation that case 4 and case 5 have similar damping which is less than the original bare case (case 1), presumably because these two cases are more miscible than case 1. We have also calculated and listed the immiscibility metric η (defined in Eq. (13) in the main text) in Supplementary Fig. 4 for the various cases. Note that simply increasing all the interaction g-parameters without notably changing η can also enhance the SDM damping, as suggested by the observation that the damping in case 3 ($\eta = -0.0045$, miscible) is stronger than that in case 1 ($\eta = -0.0045$, miscible) but is not as prominent as in case 2 ($\eta = 1.2341$, immiscible).

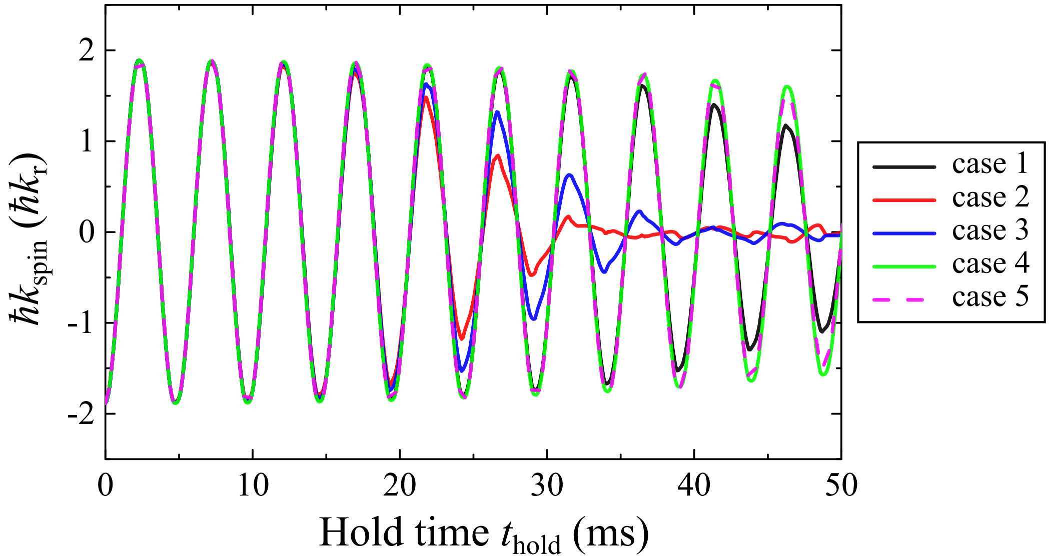

Supplementary Figure 4. **Effect of modified bare interactions and interspecies immiscibility on SDM damping.** In GPE simulations for the bare case SDM, we can change the original interaction parameters g_{ii} and g_{ij} to new values $\tilde{g_{ii}}$ and $\tilde{g_{ij}}$ respectively, where $i, j =\uparrow$ or $\downarrow$ and $i \neq j$. The relative momentum $\hbar k_{\rm spin}$ versus $t_{\rm hold}$ are shown for five different cases with the corresponding modified interaction parameters and immiscibliity metric $\eta = (\tilde{g_{\uparrow\downarrow}}^2 - \tilde{g_{\uparrow\uparrow}}\tilde{g_{\downarrow\downarrow}})/\tilde{g_{\uparrow\uparrow}}^2$ listed in Supplementary Table 1 below.

Case number	$\tilde{g_{ii}}/g_{ii}$	$\tilde{g_{ij}}/g_{ij}$	η
Case 1	1.0	1.0	-0.0045 (miscible)
Case 2	1.0	1.5	1.2341 (immiscible)
Case 3	1.5	1.5	-0.0045 (miscible)
Case 4	1.5	1.0	-0.5550 (miscible)
Case 5	1.8	1.0	-0.6896 (miscible)

Supplementary Table 1. **Cases with different modified interaction parameters and the immiscibility metric.** For each case, the corresponding immiscibliity metric $\eta = (\tilde{g_{\uparrow\downarrow}}^2 - \tilde{g_{\uparrow\uparrow}}\tilde{g_{\downarrow\downarrow}})/\tilde{g_{\uparrow\uparrow}}^2$ is calculated. The corresponding simulated SDM for each case is shown in Supplementary Fig. 4 above.

Effect of interference on the relative motion between two colliding BECs. To investigate the effect of interference on the relative motion between two colliding BECs, we have performed another set of control GPE simulations, in which two (bare) BECs are initially in a double well trap such that they are separated in real space by a potential barrier. Then, we change the double well trap to a single harmonic potential by suddenly removing the potential barrier at $t_{\text{hold}} = 0$, allowing the two BECs to collide and oscillate against each other in the y direction. We conduct the following simulations: case 1, the two BECs initially in the double well are in the same spin state (called the single spin case), with only one interaction parameter $g = \frac{4\pi\hbar^2}{m}100a_0$. Case 2, two BECs initially in the double well have orthogonal spin states ($\downarrow$ and $\uparrow$) with $g_{\uparrow\uparrow} = g_{\downarrow\downarrow} = g_{\uparrow\downarrow} = g$ (called the two spin case; here all the interaction g-parameters are set to be the same to focus on the effect of interference. The cases where the interaction g-parameters are varied differently and the effect of immiscibility are also studied separately).

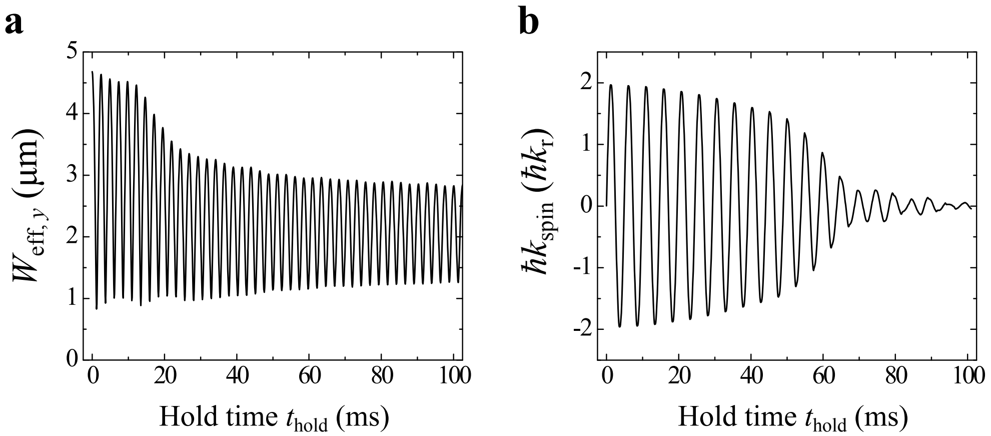

Supplementary Figure 5. **Effect of interference on the relative motion between two colliding BECs** (a) The effective width of the two BECs oscillating against each other in the y direction versus t_{hold} in the single spin case. (b) The relative momentum between the two orthogonal spin components versus t_{hold} in the two spin case. For (a, b), the two BECs are initially separated by the same potential barrier in the same double well structure. The barrier is then suddenly removed at $t_{\text{hold}} = 0$ to initiate the dynamics. Note that the oscillation frequency in (a) is twice the frequency in (b) due to the definition of $W_{\text{eff},y}$.

The damping of the relative motion in case 1 is characterized by the t_{hold}-dependent effective width ($W_{\text{eff},y}$, shown in Supplementary Fig. 5a) of the two BECs oscillating against each other in the y direction, where $W_{\text{eff},y} = \sqrt{\langle y^2 \rangle}$ ($\langle y^2 \rangle$ is the expectation value of y^2 and is calculated using the whole wavefunction of the two BECs). In this case, we find that the relative motion almost damps out after $t_{\text{hold}} = 30$ ms (when we can no longer observe any relative motion between *two* BECs, which have merged into one BEC; the relatively undamped remnant oscillations in the data after ~ 30 ms reflect the breathing of width of this merged BEC. See Supplementary Movie 1). On the other hand, in case 2 we observe prominent damping only after $t_{\text{hold}} = 60$ ms (Supplementary Fig. 5b). By comparing case 1 with case 2, we avoid the effect of immiscibility and investigate the effect purely due to the interference on damping. This suggests that the interference between the two colliding BECs can enhance the damping of the relative motion. In addition, in the two spin case we have modified the interaction parameters similar to the cases in Supplementary Fig. 4. These results also suggest that immiscibility is particularly effective to enhance the damping of SDM.

Effect of turning off interactions on the relative motion between two colliding BECs. To further investigate the role of interactions on the relative motion between two colliding BECs, we have performed three control GPE simulations where all the interaction parameters are set to zero (i.e. $g = g_{\uparrow\uparrow} = g_{\downarrow\downarrow} = g_{\uparrow\downarrow} = 0$): (1) the bare case SDM. (2) the single spin case and the two spin case with two BECs initially in a double well as described in the previous section. (3) the dressed case SDM at $\Omega_{\text{F}} = 1.3\ E_{\text{r}}$. The results of these cases are shown respectively in Supplementary Figs. 6, 7, and 8. In all these non-interacting cases, we find that the relative motion between the two colliding BECs has no noticeable damping within the time of simulation. This suggests that interactions are essential for the damping mechanisms studied in this work.

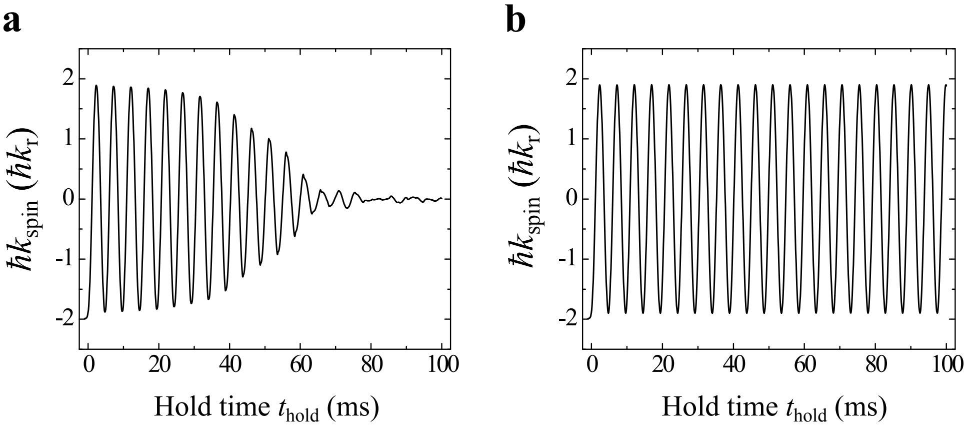

Supplementary Figure 6. **The bare case SDM with interactions in (a) and without interactions in (b).** The case (a) is the same simulation as the case of $\Omega_{\mathrm{F}} = 0$ in Fig. 6c in the main text but shown up to a longer time of 100 ms.

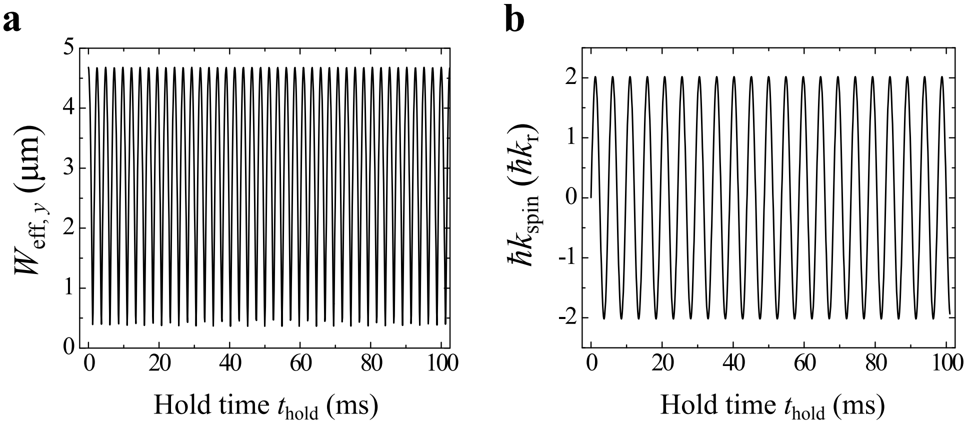

Supplementary Figure 7. **The two colliding bare BECs without interactions in the single spin case (a) and in the two spin case (b).** These simulations used the same parameters as in Supplementary Fig. 5 except the interaction g-parameters have been set to zero.

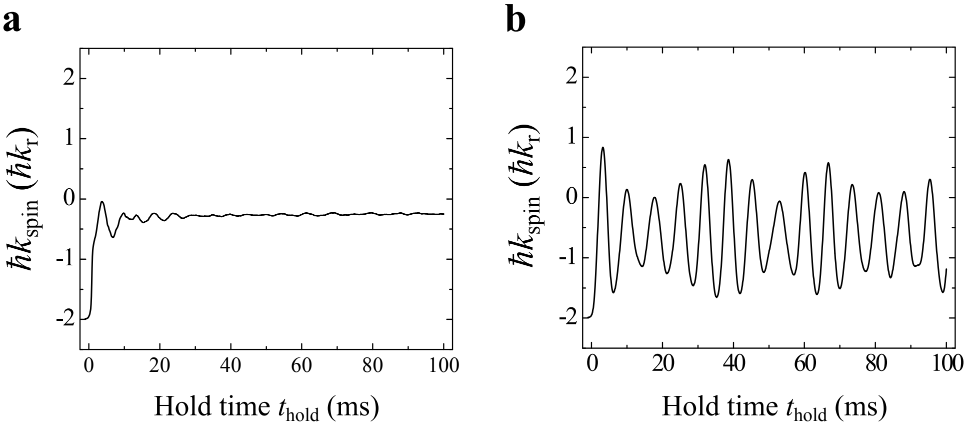

Supplementary Figure 8. **The dressed case SDM at $\mathbf{\Omega_{F} = 1.3}\boldsymbol{E_{\mathrm{r}}}$ with interactions in (a) and without interactions in (b).** The case (a) is the same simulation as the case of $\Omega_{\mathrm{F}} = 1.3\ E_{\mathrm{r}}$ in Fig. 6c in the main text but shown up to a longer time of 100 ms.

Spatial modulation in the phase of BEC wavefunctions in SDM. Supplementary Fig. 9 is an example showing the spatial modulation in the phase of BEC wavefunctions (Eq. (16) in the main text) at $t_{\rm hold} = 7.2$ ms during SDM for the bare case and the dressed case at $\Omega_{\rm F} = 1.3\ E_{\rm r}$ (snapshots taken from Supplementary Movie 3 and Supplementary Movie 6 below). We notice much less spatial variation in the gradient of the phase in the bare case than in the dressed case at $\Omega_{\rm F} = 1.3\ E_{\rm r}$, suggesting that LC KE in the bare case is generally smaller than that in the dressed case at this time (consistent with Fig. 8h in the main text).

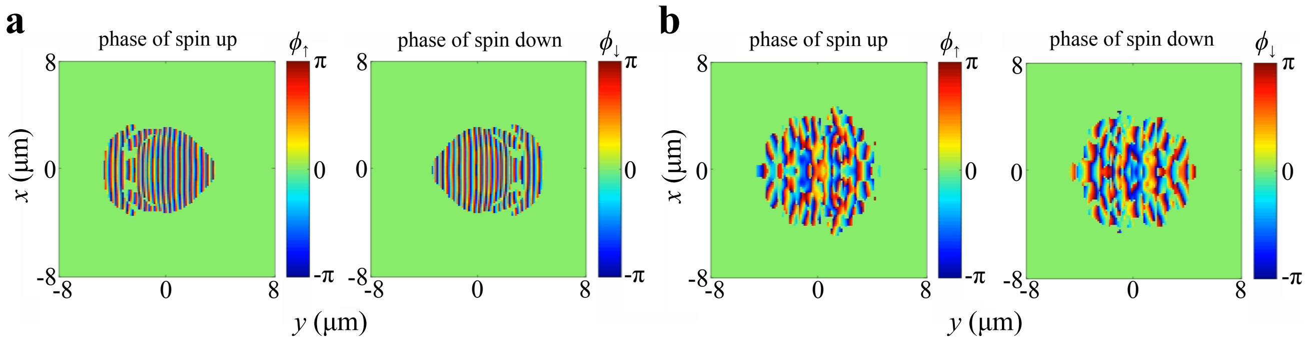

Supplementary Figure 9. **Spatial modulation in the phase of BEC wavefunctions.** The phase of the bare spin up and down components at $t_{\rm hold} = 7.2$ ms in SDM is plotted in the x-y plane for (a) bare case, and (b) dressed case at $\Omega_{\rm F} = 1.3\ E_{\rm r}$. Here, x and y are spatial coordinates.

Movies of GPE simulations for the SDM in the main text. When linked to the webpage, choose the web browser to watch it online or download the files.

Supplementary Movie 2
Supplementary Movie 3
Supplementary Movie 4
Supplementary Movie 5
Supplementary Movie 6

Here, the momentum-space (in the k_x-k_y plane) 2D density distributions (obtained by the integration over k_z, where $\hbar k_{x(y,z)}$ is the mechanical momentum in the $x(y,z)$ direction) of different bare spin components (separated vertically from each other for better visualization) are the Fourier transform of the real-space 2D densities (as those shown in Fig. 5 in the main text). The momentum-space and real-space 1D atomic densities in the y direction (SOC direction) are obtained by integrating the momentum-space and real-space 2D densities over k_x and x, respectively. In addition, the snapshot shown in Supplementary Fig. 9 for comparing the phase in the cases of $\Omega_{\rm F} = 0$ and $\Omega_{\rm F} = 1.3\ E_{\rm r}$ is taken from Supplementary Movie 3 and Supplementary Movie 6.

Supplementary Reference

[1] Zhang, J.-Y. *et al.* Collective dipole oscillations of a spin-orbit coupled Bose-Einstein condensate. *Phys. Rev. Lett.* **109**, 115301 (2012). URL `http://link.aps.org/doi/10.1103/PhysRevLett.109.115301`.
