# Supplementary material for: Spin current generation and relaxation in a quenched spin-orbit-coupled Bose-Einstein condensate
Source: Nat Commun. 2019 Jan 22;10:375. doi: 10.1038/s41467-018-08119-4 (PMC6343014; doi:10.1038/s41467-018-08119-4)
Supplement: Supplementary file 3 — Description of Additional Supplementary Information [file 41467_2018_8119_MOESM3_ESM.pdf]

Description of Additional Supplementary Files

File Name: Supplementary Movie 1
Description: The single spin case. Two BECs initially in the double well are in the same spin state.

File Name: Supplementary Movie 2
Description: SDM at $\OmegaF= 0.0$ $\E_r$: 1D and 2D atomic densities in momentum and real spaces.

File Name: Supplementary Movie 3
Description: SDM at $\OmegaF= 0.0$ $\E_r$: phase of BEC wavefunctions in real space.

File Name: Supplementary Movie 4
Description: SDM at $\OmegaF= 0.4$ $\E_r$: 1D and 2D atomic densities in momentum and real spaces.

File Name: Supplementary Movie 5
Description: SDM at $\OmegaF= 1.3$ $\E_r$: 1D and 2D atomic densities in momentum and real spaces.

File Name: Supplementary Movie 6
Description: SDM at $\OmegaF= 1.3$ $\E_r$: phase of BEC wavefunctions in real space.
