# Supplementary figures and images for: Spin current generation and relaxation in a quenched spin-orbit-coupled Bose-Einstein condensate
Source: Nat Commun. 2019 Jan 22;10:375. doi: 10.1038/s41467-018-08119-4 (PMC6343014; doi:10.1038/s41467-018-08119-4)

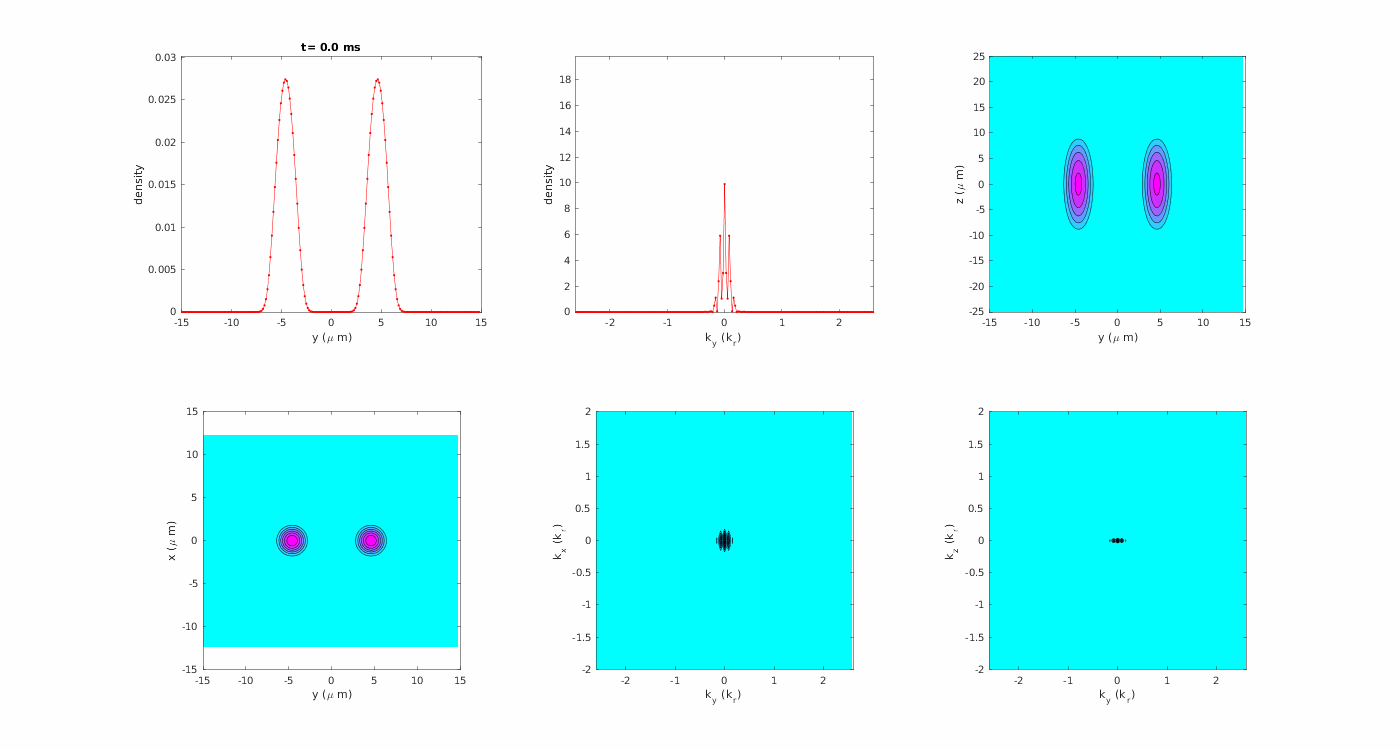

Supplement: Supplementary file 4 — Supplementary Movie 1 [file 41467_2018_8119_MOESM4_ESM.gif]

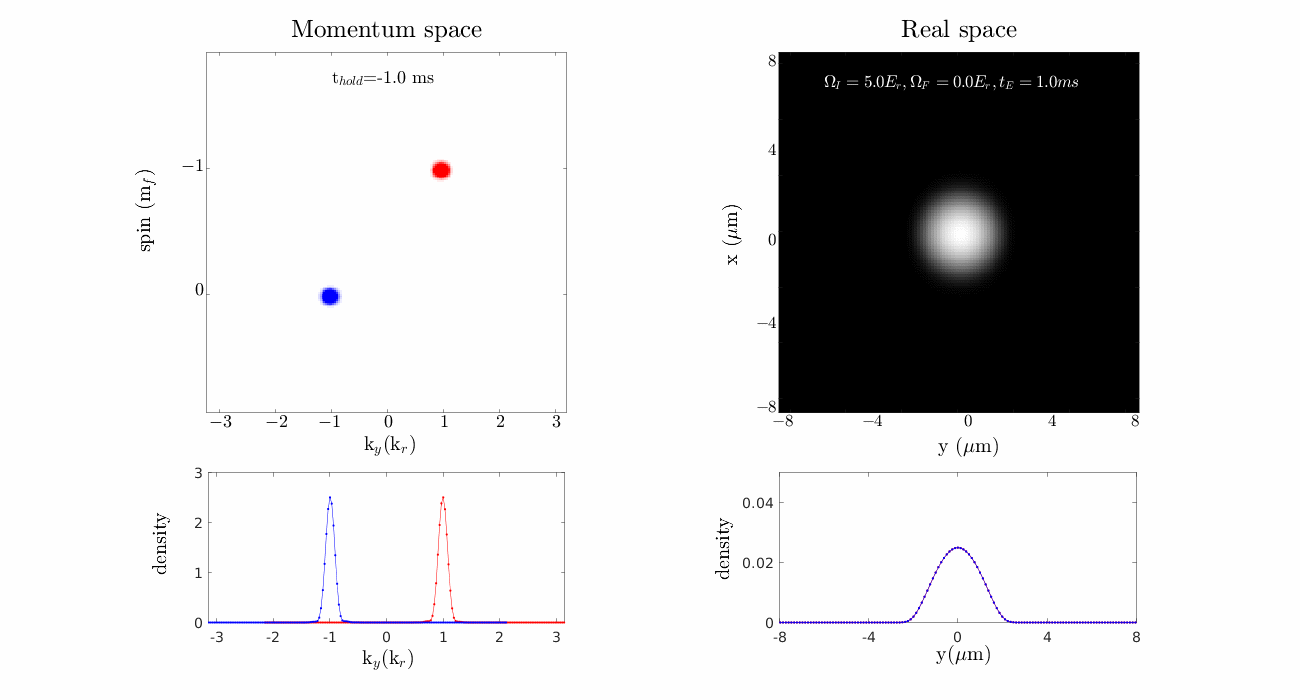

Supplement: Supplementary file 5 — Supplementary Movie 2 [file 41467_2018_8119_MOESM5_ESM.gif]

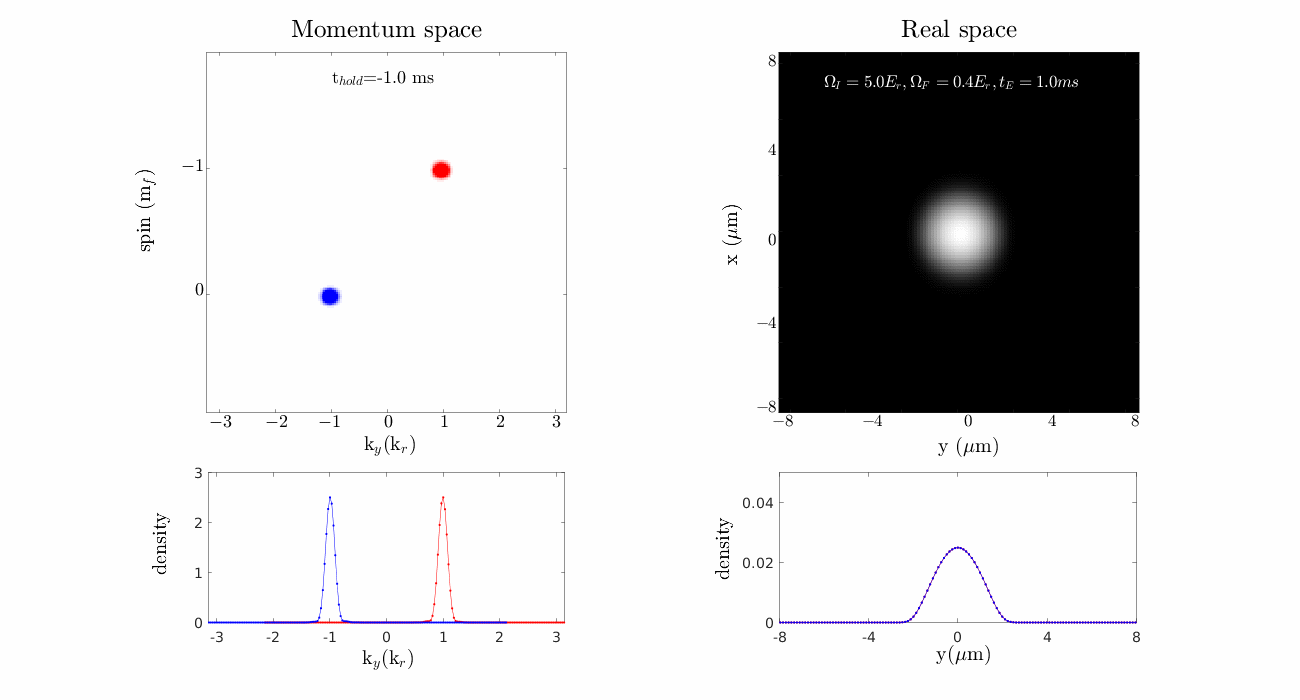

Supplement: Supplementary file 7 — Supplementary Movie 4 [file 41467_2018_8119_MOESM7_ESM.gif]

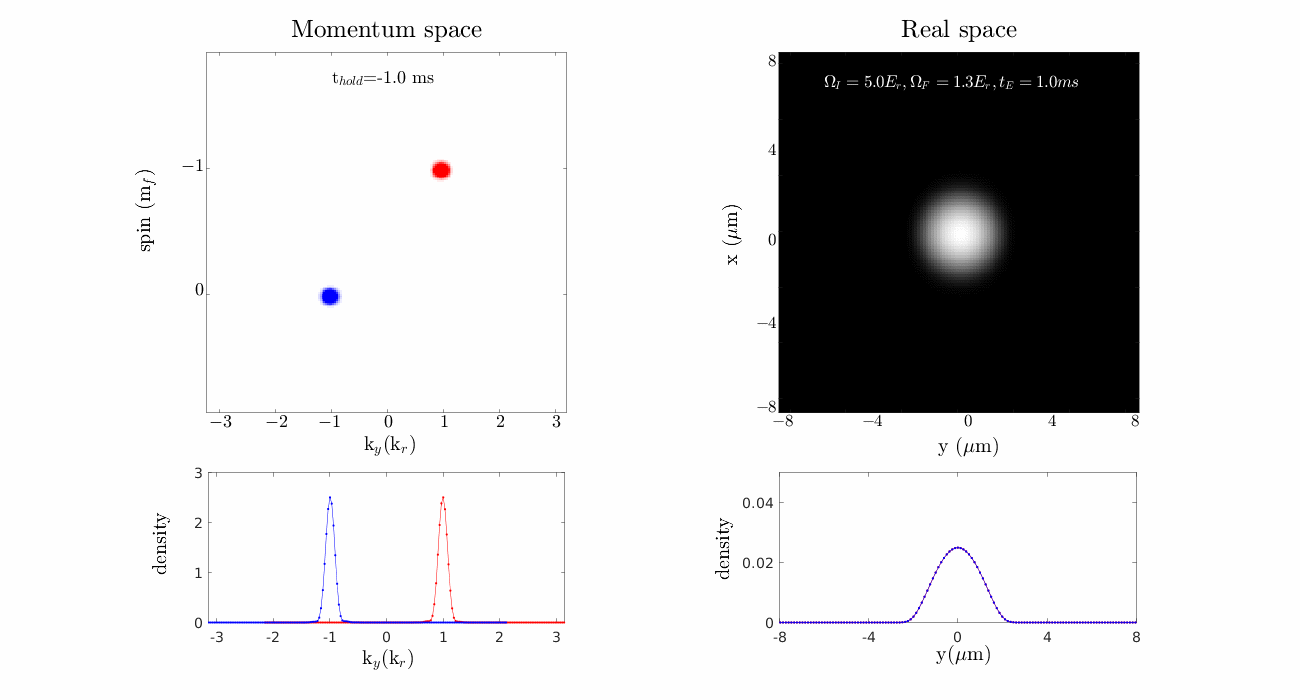

Supplement: Supplementary file 8 — Supplementary Movie 5 [file 41467_2018_8119_MOESM8_ESM.gif]
